# Amphibian skin bacteria contain a wide repertoire of genes linked to their antifungal capacities

**DOI:** 10.1007/s11274-025-04292-z

**Published:** 2025-02-27

**Authors:** Francisco González-Serrano, Yordan J. Romero-Contreras, Alberto H. Orta, M. Delia Basanta, Hugo Morales, Gabriela Sandoval García, Elena Bello-López, A. S. Escobedo-Muñoz, Víctor H. Bustamante, Víctor Ávila-Akerberg, Miguel Ángel Cevallos, Mario Serrano, Eria A. Rebollar

**Affiliations:** 1 Centro de Ciencias Genómicas, Universidad Nacional Autónoma de México, Av. Universidad s/n, Cuernavaca, Morelos 62210 México; 2 School of Environmental and Natural Sciences, Molecular Ecology & Evolution Group, Prifysgol Bangor University, Bangor, LL57 2DG UK; 3 Department of Biology, University of Nevada, Reno, 1664 N. Virginia St, Reno, NV 89557 USA; 4 Facultad de Ciencias, Universidad Nacional Autónoma de México, Av. Universidad 3000, Circuito Exterior s/n Alcaldía Coyoacán, Mexico City, Ciudad Universitaria 04510 México; 5 Facultad de Ciencias, Universidad Autónoma del Estado de México, Carretera Toluca - Ixtlahuaca Km 15.5, Piedras Blancas, Toluca de Lerdo, 50200 México; 6 Departamento de Microbiología Molecular, Instituto de Biotecnología, Universidad Nacional Autónoma de México, Apdo. Postal 510-3, Cuernavaca, Morelos 62251 México; 7 Instituto de Ciencias Agropecuarias y Rurales, Universidad Autónoma del Estado de México, Toluca, México

**Keywords:** Amphibians, Antifungal activity, *Batrachochytrium dendrobatidis*, Biosynthetic gene cluster, *Botrytis cinerea*, Chytridiomycosis, Skin microbiome

## Abstract

Emerging diseases caused by fungi are a serious threat to wildlife biodiversity. The widespread fungal pathogen *Batrachochytrium dendrobatidis* (Bd) has caused dramatic amphibian population declines and species extinctions worldwide. While many amphibians have been negatively affected by Bd, some populations/species have persisted despite its presence. One factor contributing to amphibian protection against this fungus is the host-associated skin microbiome. In this study, we aimed to identify gene clusters associated with the antifungal activity of amphibian skin bacteria. Specifically, we explored skin bacteria isolated from species that have persisted in the wild despite the presence of Bd: the frogs *Agalychnis callidryas*, *Craugastor fitzingeri*, *Dendropsophus ebraccatu*s, and the axolotl *Ambystoma altamirani*. Bacterial isolates were tested in vitro for their capacity to inhibit the growth of two fungal pathogens: Bd and the phytopathogen *Botrytis cinerea* (Bc). Genome mining of these bacterial isolates revealed a diverse repertoire of Biosynthetic Gene Clusters (BGCs) and chitin-degrading gene families (ChDGFs) whose composition and abundance differed among bacterial families. We found specific BGCs and ChDGFs that were associated with the capacity of bacteria to inhibit the growth of either Bd or Bc, suggesting that distinct fungi could be inhibited by different molecular mechanisms. By using similarity networks and machine learning, we identified BGCs encoding known antifungal compounds such as viscosin, fengycin, zwittermicin, as well as siderophores and a novel family of beta-lactones. Finally, we propose that the diversity of BGCs found in amphibian skin bacteria comprise a substantial genetic reservoir that could collectively explain the antifungal properties of the amphibian skin microbiome.

**Supplementary Information:**

The online version contains supplementary material available at 10.1007/s11274-025-04292-z.

## Introduction

Emerging fungal diseases in wildlife have become a serious threat to many animal groups worldwide (Fisher et al. 2020). In the case of amphibians, the skin disease chytridiomycosis, caused mainly by the fungus *Batrachochytrium dendrobatidis* (Bd), has led to dramatic population declines, affecting over 500 species and causing the extinction of more than 90 of them (Scheele et al. 2019). The aquatic motile zoospores of Bd penetrate the amphibian skin cells, where they mature and develop into zoosporangia, which then produce zoospores that are released into the environment (Berger et al. 2005; Rosenblum et al. 2010). The progression of Bd development inside the skin causes the tissue to lose the capacity for gas, water and electrolyte exchange, resulting in the individual’s death (Voyles et al. 2009; Rosenblum et al. 2010). Host susceptibility varies not only among species but also among populations of the same species (Smith et al. 2009; Kilburn et al. 2010; Hertz et al. 2012; Perez et al. 2014). Factors that may explain host susceptibility are the variation in host immune responses (Grogan et al. 2018), cell skin constitution (Hauser et al. 2024), and the skin microbiome, whose members can inhibit the growth of fungi directly through the production of metabolites or indirectly through competition strategies (Woodhams et al. 2015; Rebollar et al. 2020; Sentenac et al. 2024).

Although a large collection of bacteria with activity against Bd in vitro has been isolated from the amphibian skin (Woodhams et al. 2015), few antimicrobial compounds secreted by these bacteria have been described. These include the alkaloid violacein and the indole-3-carboxaldehyde produced by *Janthinobacterium lividum* (Brucker et al. 2008b), and the polyketide 2,4-Diacetylphloroglucinol (DAPG) from *Lysobacter gummosus*, both of which were isolated from the salamander *Plethodon cinereus* (Brucker et al. 2008a); the polyketide prodigiosin from *Serratia* strains, isolated from the frogs *Atelopus zeteki*, *Mantella aurantiaca* and *Alytes obstetricans* (Woodhams et al. 2018); and a cyclic lipopeptide produced by *Pseudomonas cichorii*, isolated from the skin of the frog *Lithobates warzewitschii* (Martin H. et al. 2019). These studies shed light on the chemical diversity that skin microbiomes can harbor. However, we still lack knowledge of how widespread antimicrobial molecules are across bacterial taxa isolated from different amphibian hosts.

A strategy for doing such exploration is mining the bacterial genomes from skin-derived bacterial collections searching for specialized metabolic pathways or looking for biosynthetic gene clusters (BGCs). These gene clusters are potential candidates to further explore in analytical studies in order to describe new antifungal molecules (Baltz 2021; Singh et al. 2021). Using a comparative genomic approach, recent studies have identified genes coding for a bacteriocin and a Nonribosomal peptide synthetase (NRPS) on *Pigmentiphaga sp*. isolated from the frog *Mantella crocea* (Bletz et al. 2019); nine BGCs, including DAPG in *Pseudomonas* strains from the frog *Boana prasina* (Brunetti et al. 2022); beta-lactones, aryl polyenes, and siderophores found in *Acinetobacter* strains isolated from the frogs *Agalychnis callidryas* and *Craugastor fitzingeri* (Cevallos et al. 2022); and siderophores, polyketide synthases and violacein genes coded on 40 bacterial strains isolated from amphibian species native of Eastern US (Wax et al. 2023).

The characterization of antifungal molecules and gene pathways is not only relevant to understand the symbiotic role of bacteria on the amphibian skin, but it could also yield candidates for treating infections caused by other pathogens in alternative systems. For instance, bacteria from the skin of Japanese frogs inhibit the growth of the phytopathogenic fungus *Colletotrichum orbiculare in vitro* (Susilawati et al. 2021). Bacteria from the skin of neotropical frogs inhibit the growth of Bd in vitro but also inhibited the growth of the phytopathogen *Botrytis cinerea* (Bc), which causes the gray mold disease affecting over 200 plant species, including vegetable crops, ornamental plants, and fruits (Nakajima and Akutsu 2014; Vicente-Díez et al. 2023). Thus, there is a growing interest in exploring multiple environments (like the amphibian skin) to find new biocontrol agents against Bc and other plant pathogens (Roca-Couso et al. 2021).

The exploration of antifungal capacities in amphibian skin bacteria may be particularly relevant in host species that are known to persist with Bd in their natural habitats, under the assumption that skin bacteria play a part in their protection against Bd infection (Rebollar et al. 2020). Considering the latter, we explored the antifungal capacity and the genomic content of skin bacteria isolated from four species that have persisted in their habitats despite the presence of Bd; the Neotropical frogs *Agalychnis callidryas*, *Dendropsophus ebraccatus*, and *Craugastor fitzingeri* (Rebollar et al. 2014) and the mountain stream axolotl *Ambystoma altamirani* (Basanta et al. 2023). Previous research showed that a large collection of skin bacteria, isolated from three Neotropical species, inhibited Bd growth in vitro (Rebollar et al. 2019). Moreover, metagenomic analyses have shown that the *A. altamirani* skin microbiome contains bacterial taxa with potential antifungal functions (Martínez-Ugalde et al. 2024).

In this work, we used pathogen inhibition in vitro assays and genome mining to identify potential gene candidates that could be associated with the bacterial antifungal capacity. We hypothesized that bacteria with antifungal capabilities will contain specific gene clusters that are absent in non-antifungal bacteria. The isolates used in this study were selected from a collection of bacteria derived from the skin of frogs *A. callidryas*, *D. ebraccatus*, *C. fitzingeri*, which were previously identified and tested against Bd in vitro (Rebollar et al. 2019; Cevallos et al. 2022). In addition, we constructed a new bacterial collection obtained from the skin of the axolotl *A. altamirani*, from which we assigned their taxonomy through 16S rRNA sequencing and determined their capacity to inhibit Bd growth in vitro. From both collections, we selected 67 bacterial isolates with variable growth inhibition capacities and sequenced their genomes to unravel the potential molecular mechanisms behind their antifungal phenotypes. Our findings enrich the repertoire of antifungal gene candidates derived from amphibian skin isolates. This repertoire illustrates the vast diversity of potential antifungal mechanisms present in amphibian skin microbiomes and allows us to determine potential gene candidates that could be explored in future studies.

## Materials and methods

### Bacterial collection from the skin of *Ambystoma altamirani*

From April to September of 2018, skin swab samples were obtained from the skin of *A. altamirani* individuals from a stream at Tlazala Municipality, Estado de México (exact location is not provided to protect the species from ilegal extraction). A total of 40 individuals were sampled following the procedures previously reported (Martínez-Ugalde et al. 2024) and swabs were stored in 1.5 mL microcentrifuge tubes with 1 mL of 1% Tryptone Soya Yeast Extract (TSYE) broth and 30% glycerol. The samples were kept at 4ºC during fieldwork and stored at -80 °C in the lab. Samples were thawed and 20 µl of each sample was plated onto 1% tryptone agar plates using sterile glass beads. Plates were incubated at 30 °C and monitored daily for 7 days. Morphologically distinct colonies were continuously isolated from each original plate and transferred to new 1% tryptone plates to obtain axenic cultures. All bacterial isolates were cryopreserved in 1.5 ml Eppendorf tubes with 1 ml of 1% Tryptone broth and 30% glycerol. A complete list of isolates can be found in Supplementary Table S1.

### Taxonomic identification of bacterial isolates from the skin of *A. altamirani*

Each cryopreserved bacterial isolate was thawed at room temperature, and 25µl of the liquid was plated onto a single plate with 1% tryptone agar. Plates were incubated at 30°C for 48 h or until bacterial colonies were visible. DNA for each bacterial isolate was extracted using PrepMan Ultra Sample Preparation Reagent (Applied Biosystems, Foster City, CA, USA). In the cases where DNA yields or DNA quality were low, the DNeasy Blood & Tissue Kit (QIAGEN, Inc., Valencia, CA, USA) was used. DNA was used as template for PCR reactions using specific primers to amplify the full length 16S rRNA gene: 27F (5’-AGRGTTTGATYMTGGCTCAG-3’) and 1492R (5’-GGYTACCTTGTTACGACTT-3’) (Højberg et al. 2005). The PCR protocol included: 94 °C for 4 min, followed by 94 °C for 1 min, 60 °C for 1 min, 72 °C for 1.5 min for 30 cycles, and 72 °C for 10 min. PCR products were verified using gel electrophoresis (1% agarose) and they were sent to Macrogen (South Korea) for Sanger sequencing. The reverse primer 907R (5’-CCGTCAATTCMTTTGAGTTT-3’) (Lane 1991) was used to sequence approximately 800 bp of the 16S rRNA gene for each isolate. Sequence quality analyses were done in Geneious version 6.06. Taxonomy was assigned by aligning all sequences to the SILVA release 138 database (Quast et al. 2012). All Sanger sequences were deposited in NCBI with the accession numbers: PQ656874-PQ657135.

### Bacterial selection for genomic analyses

Bacteria were chosen from a collection of isolates obtained from the skin of three tropical frog species *Agalycnhis callidryas*, *Dendropsophus ebraccatus* and *Craugastor fitzingeri* (Rebollar et al. 2019), and from the axolotl *Ambystoma altamirani* (collection obtained in this study). Based on the partial 16S rRNA sequence deposited in NCBI (accession numbers: MK506363-MK506718 for Rebollar et al. 2019 and PQ656874-PQ657135 for this study), we constructed a maximum likelihood phylogenetic tree in MEGA version 11 (Tamura et al. 2021) rooted with *Anabaena cylindrica* PCC 7122 (KM019919) as the outgroup. The tree guided our selection of isolates from distinct taxonomic classes, ensuring we had isolates that were phylogenetically close but with contrasting Bd-inhibitory capacities except for the families Xanthobacteraceae, Brucellaceae, Caulobacteraceae, Pseudomonadaceae, Enterobacteriaceae, Bacillaceae and Weeksellaceae in which all isolates from the collection had anti-Bd capacities (Fig. S1). A total of 67 isolates were selected for genome sequencing; 56 inhibited the growth of Bd in vitro and 11 did not. From this selection, nine were isolated from *D. ebraccatus*, 15 from *A. callidryas*, 19 from *C. fitzingeri*, and 24 from *A. altamirani*.

### Growth inhibition assays of the* A. altamirani* bacterial collection against *B. dendrobatidis* (Bd)

Growth inhibition activity against Bd was tested with cell-free supernatant (CFS) of each bacterial isolate following the protocol used in Cevallos (Cevallos et al. 2022). Briefly, each bacterial isolate was grown on a single tube with 1% tryptone broth for three days on a shaker (200 rpm) at 30 °C. To obtain the CFS, bacteria from each liquid culture were filtered out using a 0.22-µm filter. 50 µl of each CFS were tested in triplicate against 2 × 10^6^ Bd zoospores of the global panzootic lineage strain (JEL 423) (50 µl 1% tryptone) in 96 well plates. Bd growth was measured periodically at days 0, 4, 7, and 10 at 492 nm using a BioTek Epoch 2 Microplate Spectrophotometer (Winooski, Vermont, U.S.). 100 µl containing 2 × 10^6^ Bd zoospores growing but lacking CFS were assessed as positive growth control. To calculate the Bd growth inhibition percentage, the slope of a regression of OD_492_ readings over time was calculated, and the triplicate values from each replicate were averaged to generate a mean slope per isolate. Then, the mean slope of each isolate was divided by the mean slope of the positive control to determine the proportion of Bd growth. Finally, this proportion was subtracted from 1 to determine the inhibition score, in which the values closer to 1 represent those isolates with higher inhibition capacity. Finally, the proportions were transformed into percentages. We also employed a categorical classification that was used in further analyses: No inhibition < 20%, weak inhibition ≥ 20% and < 50%, moderate inhibition ≥ 50% and < 90%, strong inhibition ≥ 90%.

Since the Bd inhibition protocol used in this study slightly differed from the protocol used in a previous study (Rebollar et al. 2019), in which bacterial isolates for the three tropical frogs were tested, the 43 bacterial isolates derived from the frogs were re-tested to confirm their Bd inhibitory phenotypes.

### Growth inhibition assays against *Botrytis Cinerea* (Bc)

The growth inhibitory capacity of the 67 bacterial isolates against Bc was tested following a protocol previously described (Cevallos et al. 2022). Briefly, bacterial isolates were grown in LB liquid medium at 30 °C up to OD_600_ of 0.6. Then, a spore suspension of Bc B05.10 (provided by Brigitte Mauch-Mani) was prepared. Afterward, 10 µl of each bacterial isolate and 6 µl of spore suspension were placed on opposite sides of a Petri dish containing PDA medium and incubated at 24 °C for 7 days. In the control, only the fungus was used. The percentage of inhibition on Bc was calculated by measuring the radial growth of the mycelium. The diameter was measured using pictures taken by a digital camera and using the software ImageJ version 2 (NIH). Inhibition percentages were defined comparing with the total diameter of Bc in the control, averaging triplicate assays. We also defined categories to be used in further analyses: No inhibition < 18%, weak inhibition ≥ 18% and < 27%, moderate inhibition ≥ 27% and < 47%, and strong inhibition ≥ 47%.

### Sequencing, assembly, and annotation of bacterial genomes

From the 67 selected bacteria, eight genomes were previously obtained and analyzed (Cevallos et al. 2022). For the remaining 59 genomes, bacterial isolates were cultured in 5 ml of 1% tryptone broth at 30 °C and 250 rpm. Genomic DNA was extracted using two different kits: Genomic DNA purification kit (Thermo Fisher, Waltham, Massachusetts, U.S.) for Gram-negative bacteria and Qiagen DNeasy Blood and Tissue kit (Qiagen, Germantown, USA) for Gram-positive bacteria. Quantification and quality of the DNA was evaluated using NanoDrop™ and gel electrophoresis. All DNA samples were sent to Beijing Genomic Institute (China) for sequencing using the BGISEQ-2000 platform (2 × 150 bp).

The genomes were assembled using three different software: ABySS version 2.0 (Jackman et al. 2017), SPAdes version 3.9.0 (Bankevich et al. 2012), and Velvet version 1.2.10 (Zerbino and Birney 2008) with different k-mer sizes (77,87,99,121). Finally, the best assemblies were selected and improved using Metassembler version 1.5 (Wences and Schatz 2015). Genomes were checked for completeness greater than 90% and contamination or duplication using BUSCO version 5.1.2 (Manni et al. 2021) and ANVIO version 7 (Eren et al. 2020). In cases of contamination, assemblies were filtered using Maxbin version 2.0 (Wu et al. 2016). Genome assemblies generated here were uploaded to the NCBI database under BioProject ID PRJNA1200739. Additionally, genomes previously published are available with the accessions: *Acinetobacter sp. C32I*, CP098480–CP098481; *Acinetobacter sp. C26G*, CP098479; *Acinetobacter sp. C26M*, CP098478, *A. pittii A45P*, JAMOZH000000000; *A. pittii* A47H, JAMOZG000000000; *A. radioresistens C21A*, JAMOZF000000000; *A. radioresistens C30P*; JAMOZE000000000; *A. modestus C23M*, JAMOZD000000000.

### Taxonomical identification and phylogenetic inference

The taxonomic classification of bacterial genomes was determined using The Type (Strain) Genome Server version 342 (TYGS) (Meier-Kolthoff et al. 2022) which employs the German Collection of Microorganisms and Cell Cultures GmbH (DSMZ) as reference database. The server works using the dDDH metric and distances related to the GC % content. Generally, a dDDH value of 70% or higher between a new strain and a reference strain indicates that they belong to the same species (Supplementary Table S2). Finally, a genome phylogeny inference was built using PhyloPhlAn version 3.0 (Asnicar et al. 2020) which uses RAxML version 7.3.2 (maximum likelihood) with the substitution model GTR. The tree was rooted using *Anabaena cylindrica* PCC 7122 (genome reference ASM31769v1) as the outgroup.

### Identification of biosynthetic gene clusters and chitin-degrading genes

Biosynthetic gene clusters (BGCs) were identified using antiSMASH bacterial version 6 with relaxed detection strictness (Blin et al. 2019). To classify the BGC families, we used BiG-SCAPE version 1.1.3 (Navarro-Muñoz et al. 2020). BiG-SCAPE finds protein domains encoded in the BGC sequences, and uses three metrics combined in a single distance between BGCs (we used 0.7 as cutoff to assign a BGC family): (1) JI measures the percentage of shared domains, (2) AI measures the percentage of adjacent domains and (3) DSS measures the sequence identity between domains. MIBiG version 3.0 (Terlouw et al. 2023) is a database manually curated of characterized BGCs which is integrated in BiG-SCAPE software (-mibig option) and this was used to identify BGC families with previously known and experimentally tested compounds with anti-microbial functions.

Chitinases and enzymes related to chitin degradation were mined using dbCAN version 2 (Yin et al. 2012). Only gene families found with at least two algorithms were taken into account (HMMER, DIAMOND, or eCAMI). The chitin-degrading gene families (ChDGFs) were manually searched in the CAZy database and in the table of substrates from dbCAN (Substrate_05-03-2022.txt).

### Associations between BGCs and ChDGFs with bacterial taxonomy and antifungal phenotypes

In order to associate the diversity of BGCs and ChDGFs with bacterial taxonomy and with antifungal phenotypes for each fungus separately, two Euclidean distance matrices were calculated using BGC types and ChDGF abundances using vegan package (Oksanen et al. 2024). Then we performed a PCoA and permutational multivariate analysis of variance (PERMANOVA) using adonis2 function to evaluate if BGC types and ChDGF abundances varied with: bacterial family, anti-Bd phenotype, and anti-Bc phenotype considering the following comparisons: (1) fungal growth inhibition values (from 0 to 1), (2) categorical inhibition (no inhibition, weak, moderate, and strong inhibition) and (3) antifungal phenotype (inhibitory vs. non-inhibitory).

To find associations between the antifungal phenotype (anti-Bd or anti-Bc) with the diversity and abundance of BGCs and ChDGFs, three statistical tests were applied: Spearman and Phylogenetic Generalized Least Squares (PGLS) (caper version 1.03 package in R) (Orme et al. 2023) which take into account the fungal growth inhibition values, and the Mann-Whitney U test using categorical data (no inhibition, weak inhibition, moderate inhibition, and strong inhibition). We applied these tests for Bd and Bc growth inhibitory phenotypes independently. All statistical analyses and plots were made in R (R Core Team 2021).

### Identification of BGC families based on reference BGCs with known antimicrobial activity

The BGCs found on the 67 bacterial genomes, which grouped with previously characterized BGCs from MIBiG, were identified using the similarity networks built by BiG-SCAPE (Navarro-Muñoz et al. 2020). The BGC families were defined using these networks and cladograms obtained by BiG-SCAPE, and references in MIBiG were examined to determine whether the BGCs were reported as antifungal, antibacterial, anti-amoebal, or involved in metal uptake. Using this information, a putative function was assigned to each BGC family. Cytoscape version 3.9.1 (Shannon et al. 2003) was employed for network visualization.

### Prediction of antimicrobial BGCs considering the presence of antimicrobial resistance genes through machine learning

The script cluster_function_prediction.py (Walker and Clardy 2021) was employed to predict BGCs with potential antimicrobial activity using a previously trained data set and the presence of antimicrobial resistance genes coded in the bacterial genomes (Walker and Clardy 2021). Genes involved in antimicrobial resistance were identified with Resistance Gene Identifier version 4.0.3, which uses the CARD database version 3.3.0 (Alcock et al. 2019). The logic behind this strategy is that bacteria producing antimicrobial compounds would require antimicrobial resistance mechanisms to counteract self-toxicity (Walker and Clardy 2021). The support vector machine (SVM) results obtained from running the script were used for further analyses. The SVM has demonstrated better performance than the other algorithms employed in the script cluster_function_prediction.py in predicting anti-fungal and anti-eukaryotic activity (Walker and Clardy 2021). Outliers in the distribution of accuracy values for each BGC (High accuracy values) were identified from the SVM results. These outliers were then used to create similarity networks with BiG-SCAPE, and the networks were visualized in Cytoscape.

## Results

### Skin bacterial genomes display a broad repertoire of secondary metabolism genes that are linked to their taxonomy

**Fig. 1:**
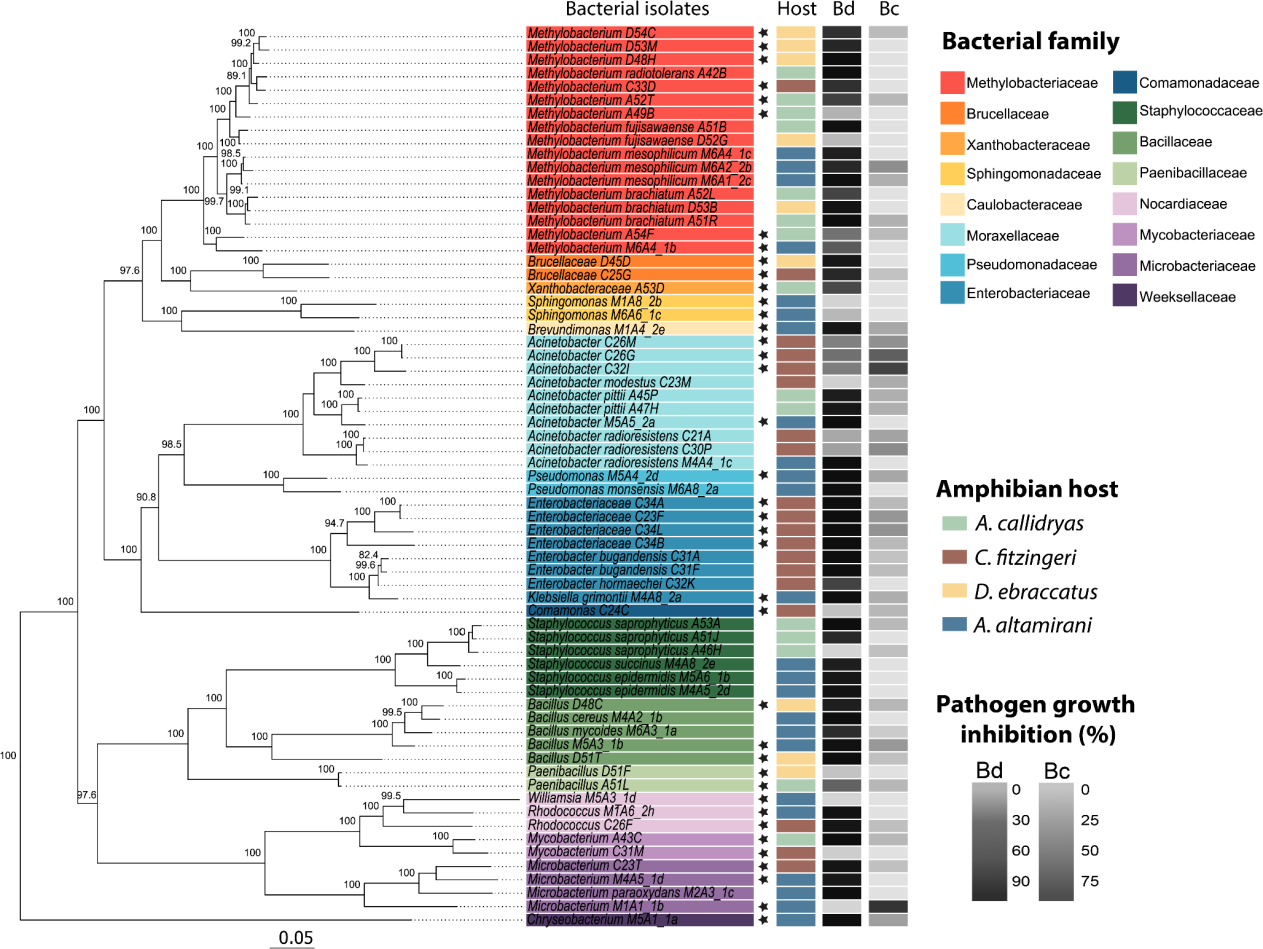
Phylogenetic tree (ML) of the 67 bacterial isolates analyzed in this study. The isolate names are highlighted with respect to their taxonomic classification at the family level. Black stars indicate genomes that could only be identified at the genus or family level. The first column next to the tree represents the host origin. The second and third columns represent the inhibition activity against Bd and Bc, respectively

We analyzed the bacterial genomes of 67 isolates: nine from individuals of *D. ebraccatus*, 15 from *A. callidryas*, 19 from *C. fitzingeri*, and 24 from *A. altamirani*. The genomes belonged to six bacterial classes, 16 families and 17 different genera. Of the 67 bacterial genomes, 30 of them could not be identified at the species level based on TYGS (Fig. 1, Supplementary Table S2).

Even though we aimed to compare isolates with contrasting phenotypes (inhibitory vs. non- inhibitory) within each bacterial family, some families were fully inhibitory against *B. dendrobatidis* (Bd), i.e. Xanthobacteraceae, Brucellaceae, Caulobacteraceae, Pseudomonadaceae, Enterobacteriaceae, Bacillaceae, and Weeksellaceae.

All the isolates whose full genome was sequenced were also tested in growth inhibition assays against the fungal phytopathogen *B. cinerea* (Bc) and the antifungal phenotypes were compared among all isolates. We found that bacteria had a variable degree of antifungal capacity against Bd (anti-Bd, mu = 73.91% inhibition, sd = 35.08%) and Bc (anti-Bc, mu = 17.65% inhibition, sd = 20.91%) and they exhibited greater anti-Bd than anti-Bc inhibitory capacity (Mann-Whitney U Test W = 4065, *p*-value = 1.436e-14) (Fig. 1). Moreover, there was not a significant association between the Bd and Bc growth inhibition capacities (Spearman rho 0.004, *p*-value 0.486, PGLS r2 0.011 *p*-value 0.393). Overall, 33 isolates inhibited the growth of both fungi and seven did not inhibit either fungus. Interestingly, a large proportion of the isolates exclusively inhibited either one of the fungi (23 inhibited Bd and four inhibited Bc), suggesting that bacteria have independent strategies for inhibiting each fungal species.

**Fig. 2:**
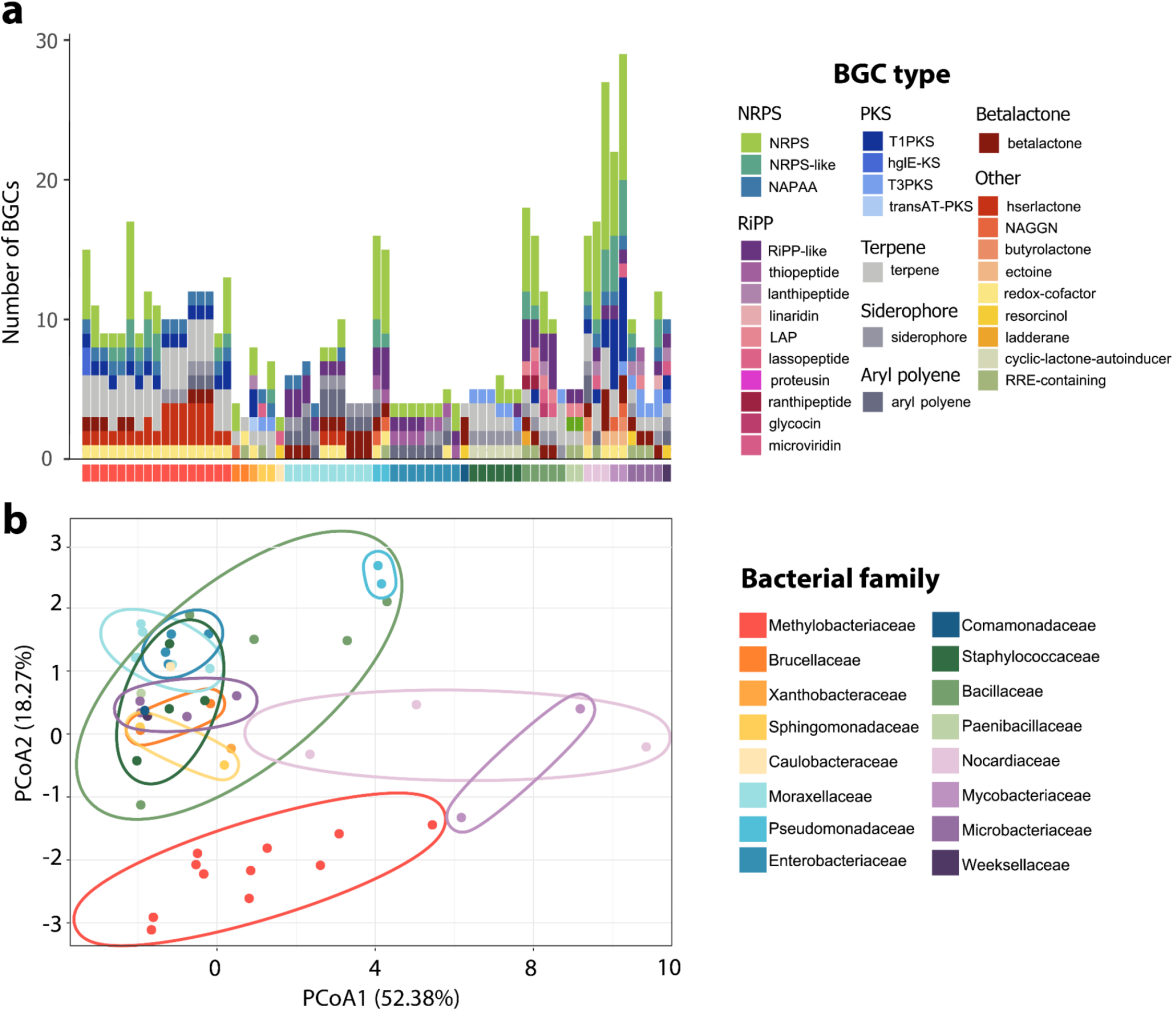
Biosynthetic gene cluster (BGC) diversity and abundance of the bacterial genomes. (**a**) Bars of different colors represent the BGC type abundance of each isolate. The X-axis indicates with colors the bacterial family for each isolate. (**b**) Principal coordinates analysis (PCoAs) of BGCs based on Euclidean distances and highlighting clusters according to bacterial family

To identify genes and/or metabolic routes that could be associated with the antifungal phenotypes, we focused on the search of Biosynthetic Genes Clusters (BGC) and chitin-degrading gene families (ChDGF) such as Glycoside Hydrolases (GH), Carbohydrate Esterases (CE), and Carbohydrate-Binding Modules (CBM). We found 30 different structural classes of BGCs grouped into eight types (Fig. 2), which included Non-Ribosomal Peptide Synthetases (NRPSs) as the most abundant (*n* = 186) followed by terpenes (*n* = 89) and Ribosomally synthesized and post-translationally modified peptides (RiPPs) (*n* = 78) (Fig. 2a, Supplementary Table S3). In addition, we found 20 different ChDGFs; the Carbohydrate-binding module CBM50 (*n* = 784) was the most abundant followed by the chitinase GH23 (*n* = 759) (Fig. S2a, Supplementary Table S3).

The diversity and abundance of BGCs and ChDGFs differed significantly among bacterial families (PERMANOVA: BGCs: r^2^ = 0.704, F = 8.092, *p* = 0.001, ChDGFs: r^2^ = 0.851, F = 19.561, *p* = 0.001) (Fig. 2b, Fig. S2b). The bacterial families Nocardiaceae and Mycobacteriaceae exhibited the largest abundance of BGCs followed by Bacillaceae and Pseudomonadaceae all of them with a broad repertory of NRPSs and Methylobacteriaceae with a large abundance of terpenes (Fig. 2a). In addition, Paenibacillaceae, Bacillaceae and Enterobacteriaceae showed the largest repertory of ChDGFs with a greater abundance of the GH18 and CBM5 families (Fig. S2a).

On the other hand, no significant differences in BGC and ChDGF diversity and abundance were found when comparing the inhibitory capacity of the isolates against Bd (Supplementary Table S4). In contrast, when analyzing the anti-Bc phenotype we found significant differences in ChDGF diversity and abundance, specifically between inhibitory vs. non-inhibitory isolates as well as with the inhibition categories (no inhibition, low, medium, and high inhibition) (In both cases PERMANOVAs: r^2^ = 0.05, *p* = 0.03) (Supplementary Table S4). However, the r^2^ values for these significant results were much lower than those obtained when comparing bacterial families (Supplementary Table S4).

### Biosynthetic gene clusters and ChDGFs of skin bacteria are associated with their antifungal phenotype

**Fig. 3:**
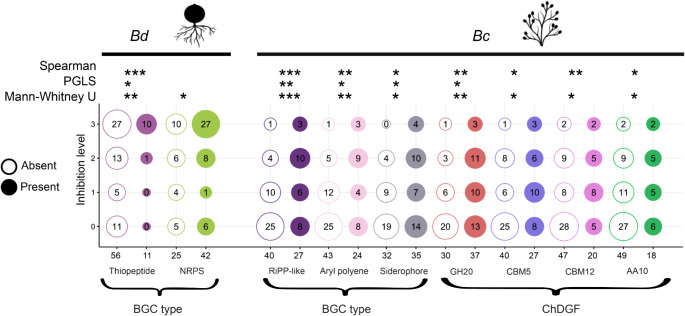
BGC types and ChDGFs significantly associated with the antifungal phenotypes using Mann-Whitney U test, Spearman, and Phylogenetic Generalized Least Squares (PGLS). The significant groups for each fungus are delimited by the black upper bars: left *B. dendrobatidis* (Bd), right *B. cinerea* (Bc). Asterisks indicate the level of significance: * = 0.05, ** = 0.01, *** = 0.001. Y-axis shows categories from low to high fungal inhibition (0 to 3) for the Mann-Whitney U test. Filled circles indicate the presence of the BGC or gene family and empty circles indicate absence. The numbers inside the circles indicate the number of isolates. Numbers at the bottom show the sum of each column

Even though BGCs and ChDGF diversity and abundance did not differ between antifungal and non-antifungal categories in almost all comparisons (Supplementary Table S4), we searched for BGC types or ChDGFs that were specifically associated with the antifungal phenotypes. For this, we employed three statistical tests: Spearman, PGLS, and Mann-Whitney U test which rely on different assumptions (see materials and methods). We found different BGC types associated with the growth inhibition for each fungus. The anti-Bd capacity was significantly associated with higher abundance of thiopeptides and NRPSs (*p*-value < 0.05) (Fig. 3, Supplementary Table S5). In contrast, the anti-Bc capacity was associated with a higher abundance of RiPP-like, siderophores, and aryl polyenes. In addition, the higher abundance of chitinase GH20, and auxiliary enzymes for chitin degradation AA10, CBM5, and CBM12 were significantly associated with the anti-Bc phenotype (Fig. 3, Supplementary Table S5).

### Some BGCs belong to families with known antimicrobial activity

**Fig. 4:**
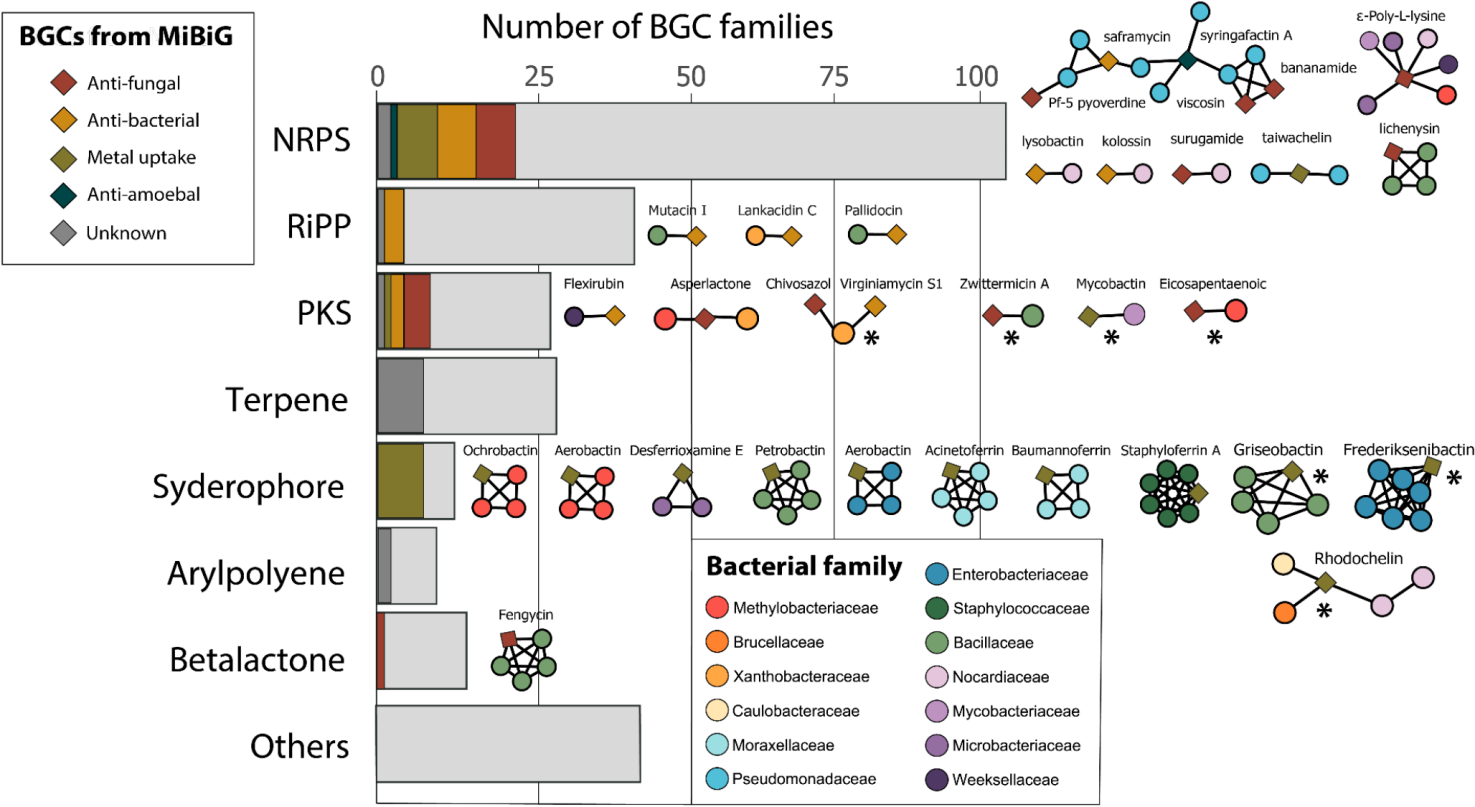
Abundance of BGC families of different types found in the bacterial genomes. Similarity networks for each BGC type are shown next to each bar. Diamonds represent the BGCs reported in MIBiG, and circles indicate the BGCs found in this study. The circles are colored according to the isolate family and the diamonds are colored according to the type of antimicrobial activity reported in the literature. Asterisks represent networks containing hybrid BGCs

To look deeper into the putative activity of the BGCs present in the bacterial genomes we grouped BGCs into families present in the MIBiG v3 database using similarity networks. We identified a total of 289 BGC families present in the bacterial genomes (Supplementary Table S6), with only 46 families (15.9% of the total) grouping with known BGCs from the MIBiG database. These families included BGCs with proven antibacterial, antifungal, antiamoebal, and metal uptake functions and were classified into seven BGC types (Fig. 4, Supplementary Table S7). Notably, NRPSs, polyketide synthases (PKSs), and RiPPs were the BGCs with a higher number of antifungal and antibacterial activities. The bacterial families with more BGCs linked to antifungal and antibacterial activities were Methylobactericeae, Nocardiaceae, Bacillaceae, Xanthobactereceae, and Pseudomonadaceae (Fig. S3).

We found that most of the BGC family networks are composed of members from a single bacterial family, with the exception of ε-Poly-L-lysine, rhodochelin, and asperlactone networks (Fig. 4). Networks with characterized antifungal compounds were the fengycin (Tao et al. 2011) and lichenysin (Guo et al. 2023), both found in Bacillaceae, and viscosin (Martin H. et al. 2019) and pyoverdine (Sass et al. 2021) found in Pseudomonadaceae. We also observed a large variety of siderophores that were present in particular bacterial families: frederiksenibactin in Enterobacteriaceae, acinetoferrin in Moraxellaceae, and staphyloferrin A in Staphylococcaceae (Fig. 4, Supplementary Table S7). Interestingly, with the exception of mycobactin and ε-Poly-L-lysine all other networks exclusively included BGCs present on the antifungal isolates, suggesting that these BGCs could be associated with their antifungal capacity (Fig. S3, Supplementary Table S7).

### Some BGCs from antifungal isolates are predicted to have antifungal activity

Given the limited information available on the functions of BGCs (Fig. 4), we employed a support vector machine (SVM) algorithm that has been previously used for predicting antimicrobial BGCs (Walker and Clardy 2021). From the genomes of Bd-inhibitory isolates, we found 26 BGCs with high accuracy values of antifungal activity (outliers in the distribution of accuracy values) in comparison to only two BGCs from non-Bd inhibitory isolates (Fig. S4a, Supplementary Table S8). These correspond to nine NRPS, seven beta-lactone, six terpenes, two NRPS/PKS, one NRPS/RiPP, and one aryl polyene. Eight of these BGCs belong to previously identified antifungal BGC families (Fig. S4a, Supplementary Table S8) bananamide (Omoboye et al. 2019), lichenysin (Guo et al. 2023), chivosazol (Irschik et al. 1995), zwittermicin A (He et al. 1994), and ε-Poly-L-lysine (Purev et al. 2020), the anti-amoebal family syringafactin A (Zhang et al. 2021) and the antibacterial family virginiamycin S1 (Pulsawat et al. 2007). In addition, when analyzing predicted anti-eukaryotic activity, we found 21 BGCs with high accuracy values in Bd-inhibitory isolates in comparison to only one BGC from a non-Bd inhibitory isolate (Fig. S4b, Supplementary Table S8). These BGCs belonged to 14 beta-lactones, three aryl polyenes, two NRPS, and two NRPS/PKS. (Supplementary Table S8). When comparing these BGCs to the MiBiG database two of them belonged to the antifungal BGC families zwittermicin A (He et al. 1994) and chivosazol (Irschik et al. 1995), and the antibacterial BGC family virginiamycin S1 (Pulsawat et al. 2007) (Fig. S4b), all of them also predicted with the anti-fungal algorithm. In contrast, Bc-inhibitory isolates showed a similar number of predicted antimicrobial BGCs to the non-Bc inhibitory isolates (Fig. S4c-d).

**Fig. 5:**
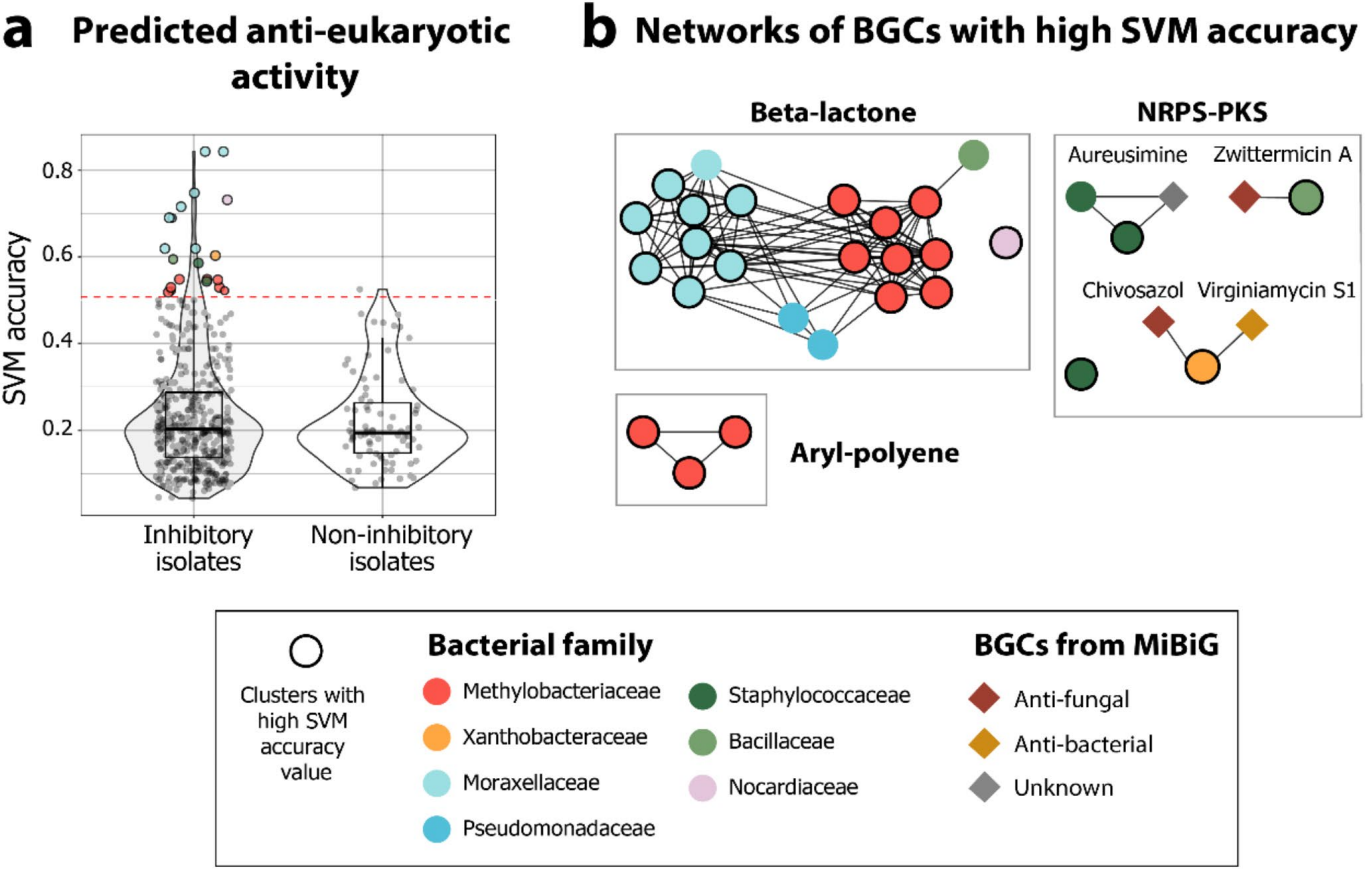
Prediction of anti-eukaryotic activity using Support Vector Machine (SVM) (**a**) SVM accuracy values of predicted anti-eukaryotic activity of BGCs present in all bacterial isolates (Y-axis). The X-axis separates the distribution of Bd inhibitory isolates and non-inhibitory isolates. The dots above the red line are the outliers. (**b**) Similarity networks of BGCs with high SVM accuracy values separated by BGC family. Circular nodes represent BGCs from the isolates; those with black circumferences were identified as outliers in Fig. 5a. The nodes are colored based on the bacterial family. Diamonds represent BGCs from the MiBiG database colored according to the type of antimicrobial activity

Finally, we used similarity networks to identify how the BGCs, predicted to have anti-eukaryotic activity in the Bd-inhibitory isolates (Fig. 5a), were interconnected among themselves and with the rest of the BGCs reported in MIBiG (Fig. 5b). We identified a network of aryl-polyene coded in methylobacteriaceae isolates; four NRPS-PKS type networks, one containing two Staphylococcaceae grouped with aureusimine, another network of Bacillaceae grouped with Zwittermicin A, a BGC from Xanthobactereaceae grouped with chivosazol and virginiamycin S1, and a single BGC coded in a Staphylococcaceae isolate. Moreover, we found a BGC network of beta-lactones present only in Moraxellaceae anti-Bd isolates along with BGCs belonging to Pseudomonadaceae and Methylobacteriaceae (Fig. 5b).

## Discussion

To date, thousands of bacterial strains capable of inhibiting Bd have been isolated from the amphibian skin microbiome (Woodhams et al. 2015; Rebollar et al. 2020). However, few studies have focused on uncovering the genetic bases behind this antifungal capacity (Woodhams et al. 2018; Cevallos et al. 2022; Wax et al. 2023; Martínez-Ugalde et al. 2024). Moreover, recent studies have shown that bacteria derived from the amphibian skin can be a source of biocontrol agents against plant pathogens (Susilawati et al. 2021; Romero-Contreras et al. 2024a).

Here, we explored bacterial genomes derived from the amphibian skin and found biosynthetic gene clusters (BGCs) and chitin-degrading gene families (ChDGFs) associated with the capacity of bacteria to inhibit pathogenic fungi. One limitation of our study is that we did not test whether these genes are actively expressed nor whether their bioactive compounds or enzymes are responsible for the observed antifungal phenotype. Thus, further investigations are essential to validate these associations, employing transcriptomic or metabolic data, as well as molecular genetic techniques that allow us to identify specific targets (Baltz 2021; Singh et al. 2021). In sum, our predictions could serve as a guide for further exploring the mechanisms behind the antifungal activity of the skin microbiome and for characterizing novel biological control agents.

### Antifungal capacity of skin bacteria is linked to their BGCs and ChDGFs

We found significant associations between the presence/abundance of specific BGC types and ChDGFs, and the antifungal capacity of the isolates (anti-Bd or anti-Bc), even when controlling for phylogenetic distances among them. Specifically, thiopeptides, Ribosomally synthesized and post-translationally modified peptides-like (RiPP-like) and Nonribosomal peptide synthetases (NRPS), were associated with the anti-Bd phenotype. These BGCs have been previously linked to antibacterial and antifungal functions (Mizuhara et al. 2011; Xuan et al. 2023; Pfeiffer et al. 2024). For instance, they are involved in pathways synthesizing cyclic and macrocyclic peptides (Mordhorst et al. 2023) which are implicated in the destabilization of the membrane integrity of pathogens (Helmy and Parang 2023). Other mechanisms include inducing cell-wall fragility by binding to chitin, and synthesis inhibition of cell-wall components (Helmy and Parang 2023).

We found an association between the Bc-inhibitory phenotype and RiPP-like, aryl polyenes and siderophores, as well as specific ChDGFs: GH20, CBM5, CBM12, AA10. Aryl polyenes are widespread metabolites (pigments) found in Gram-negative bacteria involved in protection against oxidative stress (Schöner et al. 2016) and linked to immune system evasion and biofilm formation (Cimermancic et al. 2014; Lee et al. 2021; Johnston et al. 2021). More work is needed to determine their potential role as antifungal molecules. On the other hand, it is known that siderophores main function is harvesting metals from the environment (Ahmed and Holmström 2014). Thus, siderophores can be part of a competitive strategy to avoid the establishment or colonization of other microorganisms including fungi (Bachman and Weiser 2015; Khan et al. 2021). Gene families (GH20 and GH23) and CBMs (responsible for substrate recognition) could be used in conjunction to efficiently degrade chitin. Chitin is one of the main compounds in fungal cell walls (Veliz et al. 2017), including *Bc* structures like the conidia and the mycelium (Cantu et al. 2009). Moreover, bacteria producing chitinolytic enzymes have been shown to inhibit the growth of plant fungal pathogens (Hamid et al. 2013; Langner and Göhre 2016; Lu et al. 2021). In contrast, we did not find ChDGFs that were significantly associated with Bd growth inhibition. This is consistent with the fact that Bd zoospores lack a cell wall of chitin (Silva et al. 2019) and thus chitin degradation would not be a trait associated with the anti-Bd phenotype.

### Genome mining of antifungal bacteria can guide the search for novel antifungal targets

The exploration of amphibian skin bacterial genomes evidenced the large gap of knowledge that exists when analyzing new bacterial genomes. From the 289 families of BGCs present in our bacterial collection, only 46 have been previously characterized. This result indicates that the vast diversity of BGCs found in this study are novel, thereby representing a source with high potential for the identification of possible new compounds with antimicrobial activity.

When using similarity networks to associate the BGCs present in our bacterial collection with known BGC families, we found a large repertoire of clusters with previously reported antifungal activity such as viscosin (Martin H. et al. 2019), bananamide (Omoboye et al. 2019), syringafactin A (Zhang et al. 2021), pyoverdine (Sass et al. 2021), fengycin (Tao et al. 2011), chivosazol (Irschik et al. 1995), zwittermicin A (He et al. 1994) and asperlactone (Gonçalves et al. 2021). Specifically, viscosin-like molecules produced by *Pseudomonas cichorii* have been previously associated with antifungal activity against Bd (Martin H. et al. 2019). We also found BGC families coding for previously characterized siderophores such as Frederiksenibactin (Stow et al. 2021) and acinetoferrin (Funahashi et al. 2013). Importantly, the majority of the clusters with known antimicrobial activity were found in bacterial isolates with antifungal capacities. This suggests that these clusters could be responsible for the observed phenotype in vitro highlighting them as promising candidates for further investigations.

When using machine learning predictions, we found additional BGCs such as beta-lactones predicted to have anti-eukaryotic activity. These BGCs were found in all Moraxellaceae isolates that showed anti-Bd activity. Beta-lactones are compounds that have received significant attention because of their antifungal capacities (Robinson et al. 2019). Their targets include important enzymes such as lipases, proteases, esterases, and fatty acid synthetase thioesterases (Robinson et al. 2019). Thus, future studies are needed to determine if the beta-lactone cluster found here indeed codes for compounds with antifungal activity. Such investigations could provide valuable insights into the mechanisms of action and potential applications of the bioactive compounds.

### Antifungal functions as part of the ecological interactions occurring within the amphibian skin microbiome

The protective function associated with the amphibian skin microbiome is likely an emerging property of the interactions occurring within the microbial community (Geesink et al. 2024). In this respect, the genetic repertoire found on skin bacteria, along with their antifungal activity, showcases the diverse mechanisms that they use to compete and survive in the skin/mucous environment, which is also modulated by the host (Smith et al. 2023; Wilde et al. 2024).

The presence of unique genetic signatures associated with amphibian skin bacteria has been observed in previous comparative genomics studies. For example, *Pigmentiphaga* strains have been found in the skin of frog species from geographically distant locations such as Madagascar and Panama, and these isolates exhibit different genetic repertoires compared to *Pigmentiphaga* strains isolated from soil (Bletz et al. 2019). Similarly, *Pseudomonas* strains isolated from frogs harbored NRPSs with predicted antifungal activity, which were absent in *Pseudomonas* strains from plants and other animals (Brunetti et al. 2022). These findings suggest that amphibians may share mechanisms for selecting specific bacteria with specialized genetic repertoires.

In this study, we found bacterial isolates (with a similar composition of BGCs and ChDGFs) derived from amphibian species with distinct lifestyles and/or geographic origin, including tropical arboreal species like *A. callidryas* and *D. ebraccatus*, tropical terrestrial species like *C. fitzingeri*, and aquatic species like *A. altamirani* living in streams of temperate forests. While the specialized genetic repertoire is conserved at the bacterial family level, it is very interesting that these bacteria can be found across different amphibian species, such as *Methylobacterium* and members of the *Moraxellaceae* family. This could imply that these bacteria are widely selected within amphibian skin microbiomes and are specifically adapted to inhabit the amphibian skin.

The production of secondary metabolites (Brucker et al. 2008a, b; Woodhams et al. 2015; Martin H. et al. 2019) or biofilm formation (Sentenac et al. 2024) are some of the broad mechanisms involved in pathogen protection in amphibians. For instance, many compounds produced by amphibian skin bacteria (Woodhams et al. 2018) are effective in combating fungi and bacteria such as violacein (Durán et al. 2022) and prodigiosin (Yip et al. 2021). Here, we identified a broad repertoire of BGCs and ChDGFs that may exert these broad antimicrobial effects. Moreover, we also found specific BGCs and ChDGF that were significantly associated with either anti-Bd or anti-Bc phenotypes, suggesting that some pathways may be involved in specialized responses towards different fungal lineages (chytridiomycota vs. Ascomycota). Further studies are needed to determine the interactions occurring among bacterial isolates and whether these anti-fungal effects remain within skin communities in a natural setting.

### Bioprospection of amphibian skin bacteria may benefit wildlife conservation and agriculture

There have been attempts to use anti-Bd bacteria as probiotic treatments for chytridiomycosis (Kueneman et al. 2016; Harrison et al. 2020; Becker et al. 2021). However, few studies have explored the genetic mechanisms behind the antifungal capacity of members of the amphibian skin microbiome (Martin H. et al. 2019, Brunetti et al. 2022; Cevallos et al. 2022; Wax et al. 2023; Martínez-Ugalde et al. 2024). Understanding this genetic basis is essential for a broader comprehension of the interactions between amphibian skin-associated bacteria and Bd, and may be relevant to propose effective treatments against this pathogen.

Furthermore, the potential applications of amphibian skin bacteria extend beyond amphibian conservation. There is promising evidence that these bacteria can be used in agriculture as plant growth promoters by stimulating the production of phytohormones (Susilawati et al. 2021; Romero-Contreras et al. 2024b) or as fungal pathogen inhibitors (Susilawati et al. 2021; Romero-Contreras et al. 2024a). Future studies should include in vivo experiments and field trials to confirm the effectiveness of these potential biocontrol agents under natural conditions. Additionally, comprehensive analyses of the bioactive compounds, including chemical characterization and direct antifungal activity assessments, as well as synergistic interaction studies, should be considered. We encourage the investigation of these isolates and their bioactive compounds in the pursuit of solutions for wildlife conservation and for agriculture improvements.

## Electronic supplementary material

Below is the link to the electronic supplementary material.

Supplementary Material 1

Supplementary Material 2

## Data Availability

16s rRNA Sanger sequences from *A. altamirani* were deposited in NCBI with the accession numbers: PQ656874-PQ657135. Genome assemblies generated here were uploaded to NCBI database under BioProject ID PRJNA1200739.
