# Supplementary material for: Amphibian skin bacteria contain a wide repertoire of genes linked to their antifungal capacities
Source: World J Microbiol Biotechnol. 2025 Feb 27;41(3):78. doi: 10.1007/s11274-025-04292-z (PMC11865118; doi:10.1007/s11274-025-04292-z)
Supplement: Supplementary file 1 — Supplementary Material 1 [file 11274_2025_4292_MOESM1_ESM.docx]

**Amphibian skin bacteria contain a wide repertoire of genes**

**linked to their antifungal capacities**

World Journal of Microbiology and Biotechnology

Francisco González-Serrano^1^, Yordan J. Romero-Contreras^1^, Alberto H. Orta^1,2^, M. Delia Basanta ^3,4^, Hugo Morales^5^, Gabriela Sandoval García^4^, Elena Bello-López^1^, A. S. Escobedo-Muñoz^1^, Víctor H. Bustamante^6^, Víctor Ávila-Akerberg^7^, Miguel Ángel Cevallos^1^, Mario Serrano^1^, Eria A. Rebollar^1*^.

1 Centro de Ciencias Genómicas, Universidad Nacional Autónoma de México, Av. Universidad s/n, Cuernavaca, 62210, Morelos, México.

2 School of Environmental and Natural Sciences, Molecular Ecology & Evolution Group, Prifysgol Bangor University, Bangor, LL57 2DG, UK.

3 Department of Biology, University of Nevada Reno, 1664 N Virginia St, Reno, 89957, NV, USA.

4 Facultad de Ciencias, Universidad Nacional Autónoma de México, Mexico City, México.

5 Facultad de Ciencias, Universidad Autónoma del Estado de México, Carretera Toluca - Ixtlahuaca Km 15.5, Piedras Blancas, 50200 Toluca de Lerdo, México.

6 Departamento de Microbiología Molecular, Instituto de Biotecnología, Universidad Nacional Autónoma de México, Apdo. Postal 510-3, Cuernavaca, Morelos 62251, México.

7 ​Instituto de Ciencias Agropecuarias y Rurales, Universidad Autónoma del Estado de México, Toluca, México.

*Corresponding author: Eria A. Rebollar

E-mail: [rebollar@ccg.unam.mx](mailto:rebollar@ccg.unam.mx)

**SUPPLEMENTARY FILE 1**

1. **Supplementary Figures**

**Fig. S1.** Phylogenetic tree (ML) of the bacterial isolate collection from four amphibian species (Rebollar et al. 2019, this work).

**Fig. S2.** Chitin-degrading gene family (ChDGF) diversity and abundance of the amphibian skin isolate collection.

**Fig. S3.** BGC families with antimicrobial members and their associated taxonomy.

**Fig. S4.** Prediction of antimicrobial activity of BGC using Supervised Vector Machine (SVM).

1. **Supplementary Tables**

**Table S1.** General information of the *A. altamirani* isolate collection. Column A is the ID of the isolate, column B is the accession number from NCBI, columns C to H show the taxonomic classification using 16S: phyla, class, order, family, genus and species. Column I contains Bd growth inhibition percentages and column J indicates if the genome was sequenced.

**Table S2.** General information of the isolates selected for genome sequencing. Column A indicates the ID assigned to each isolate, column B the host, column C the assigned name based on 16S and genome sequences comparisons, column D the taxonomy, columns E-U include genome metrics and columns V-AA include metrics associated with the bacterial inhibition capacity against *B. dendrobatidis* (Bd) and *B. cinerea* (Bc).

**Table S3.** BGC types and ChDGFs abundance per bacterial isolate.

**Table S4.** Permutational multivariate analysis of variance (PERMANOVA) table showing the significant effect of antifungal inhibition and bacterial family on BGC type and ChDGF diversity. The number of permutations employed was 999.

**Table S5.** Association between BGC types and ChDGF abundances and isolate inhibitory activity. For Mann-Whitney U tests the inhibition categories were used and for Spearman and Phylogenetic generalized least squares (PGLS) the inhibition growth percentages were used (see materials and methods).

**Table S6.** Number of families for different types of BGCs. Families are classified using Big-SCAPE (c=0.7). Subtypes were included within their types, and in Others were included the remaining BGC types (beta-lactones, siderophores, aryl polyenes, among others).

**Table S7.** BGCs from MIBiG grouped into families with BGCs from the isolates. Column A indicates the ID from my MIBiG, column B the compound name synthesized by the BGC, column C the BGC family number assigned by Big-SCAPE (c=0.7), column D the family of the isolates with a similar BGC, column E the antifungal isolate IDs containing the BGC, column F the non-antifungal isolate IDs containing the BGC, column G the BGC IDs, column H the MIBig BGC type, column I the BiG-SCAPE/antiSMASH class, column J the type of activity found in literature and column K the reference where the activity was described.

**Table S8.** Support Vector Machine (SVM) accuracy values for BGCs present in the bacterial genomes. Column A represents the ID of the BGC, column B the ID of the isolate, column C the BGC type, column D the family number assigned by Big-SCAPE (c=0.7), column E the BGC match from MIBiG, column F the name of the match, column G the proposed activity for the BGC, columns H-I the accuracy of the SVM algorithm of having anti-eukaryotic and antifungal activity, column J the name of the isolate, columns K-P the isolate inhibition activity against *B. dendrobatidis* (Bd) and *B. cinerea* (Bc).

**SUPPLEMENTARY FIGURES**


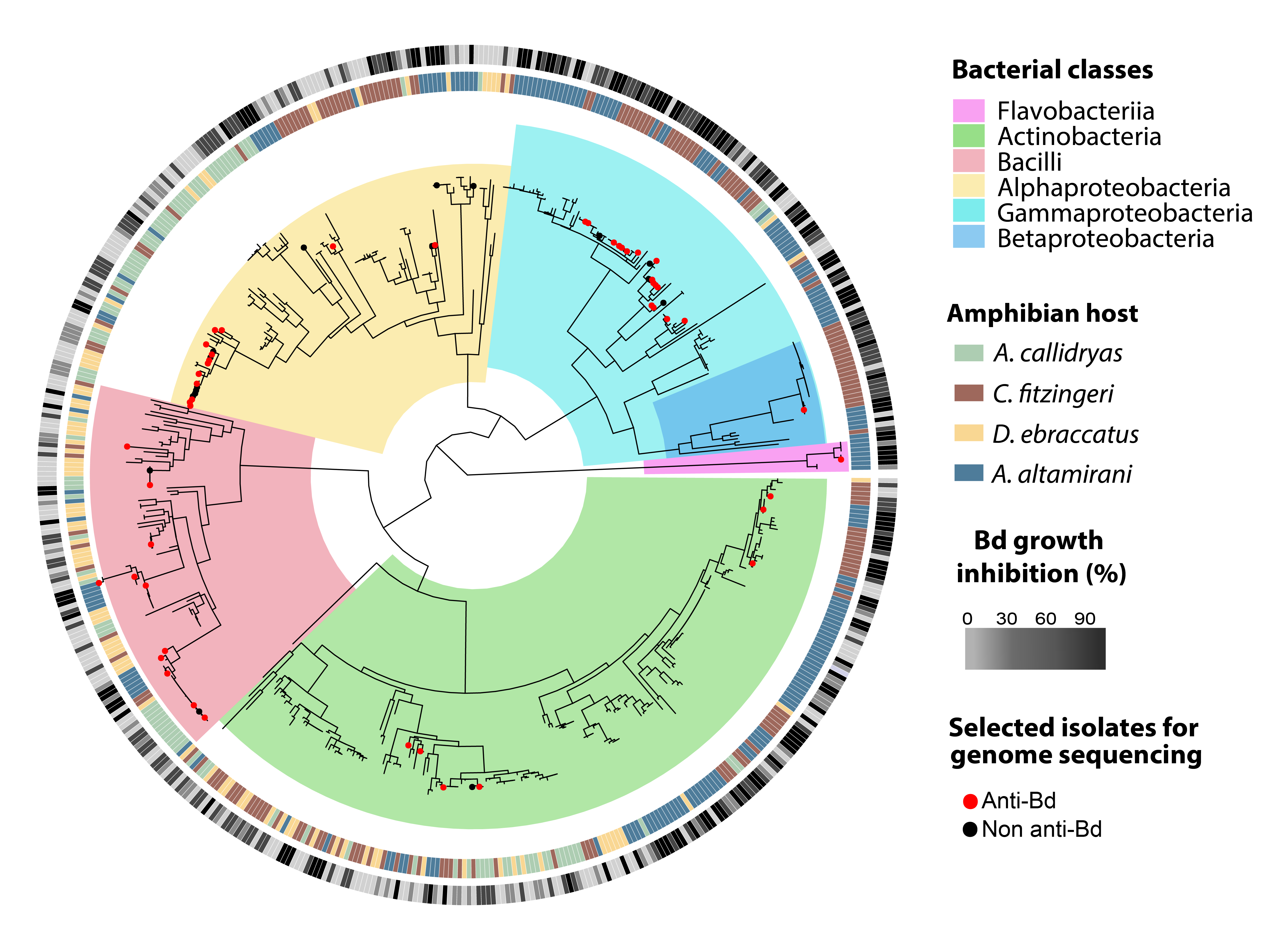


**Fig. S1.** Phylogenetic tree (ML) of the bacterial isolate collection from four amphibian species (Rebollar et al., 2019 and this work). Taxonomic classes of bacteria are shown in different colors within the phylogenetic tree. Circles on the tips of the branches represent the bacteria selected for genome sequencing with anti-Bd activity (red circles) and non-anti-Bd activity (black circles). Amphibian host is shown on the inner circle, and Bd inhibitory capacity is presented on the outer circle.


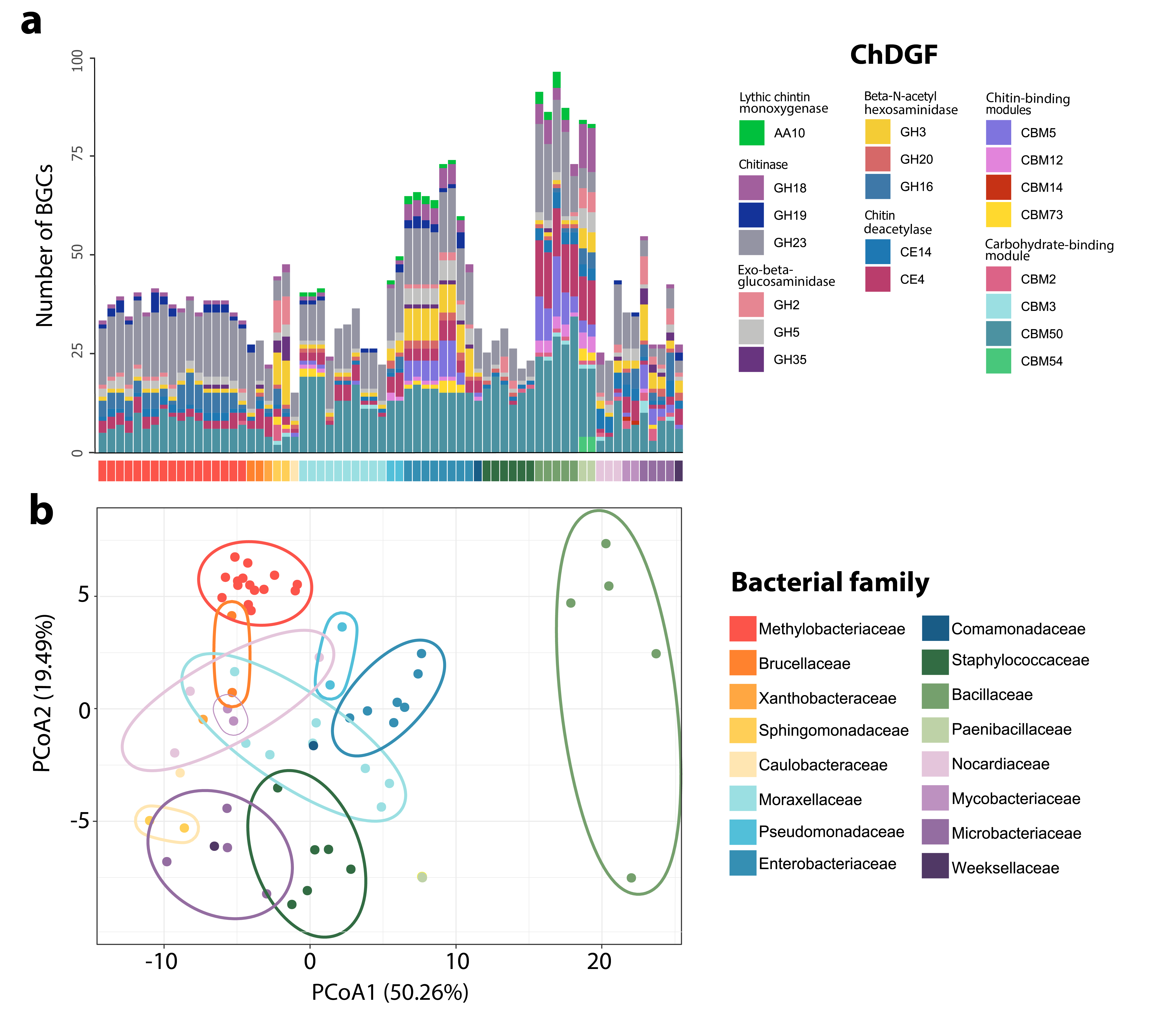


**Fig. S2.** Chitin-degrading gene families (ChDGF) diversity and abundance of the amphibian skin isolate collection. (a) Bars with different colors represent the ChDGF abundance of each isolate. X-axis shows the bacterial family of each isolate in different colors. (b) Principal Coordinates Analysis (PCoAs) of ChDGF based on Euclidean distances.


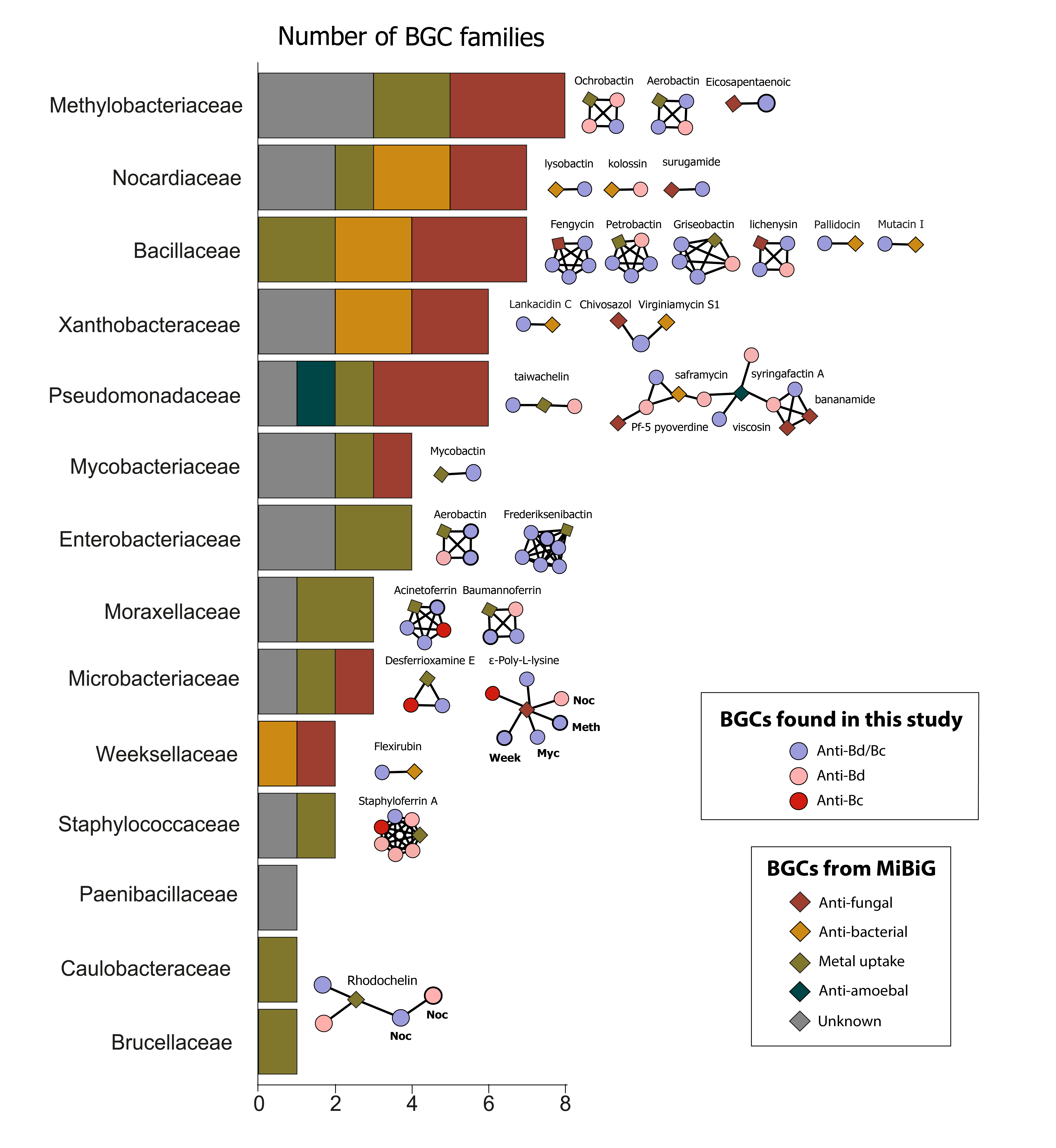


**Fig. S3.** BGC families with antimicrobial members and their associated taxonomy. Bars correspond to the number of families of each different BGC type per bacterial family. Similarity networks contain BGCs previously characterized with antimicrobial activity and the isolates’ antifungal phenotypes. Diamonds represent the BGCs characterized from MIBiG and circles the BGCs from the isolate collection. Circles are colored according to the isolate antifungal phenotype and diamonds according to the type of antimicrobial activity reported in the literature.


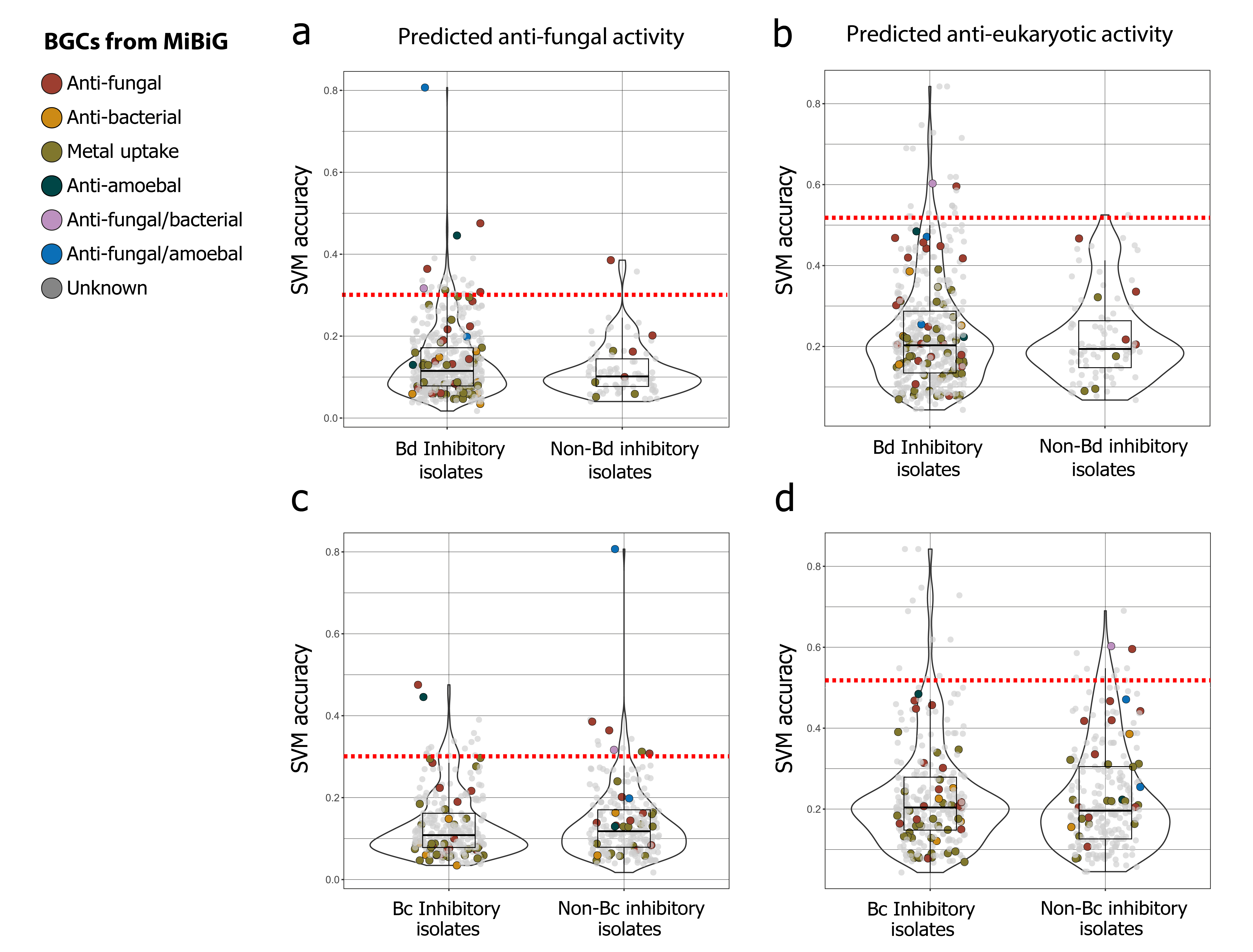


**Fig. S4**. Prediction of the BGC activities with Supervised Vector Machine (SVM). Distributions of antifungal (a, c) anti-eukaryotic (b, c) activity of BGCs coded in inhibitory isolates and non-inhibitory isolates against Bd (a, b), and Bc (c, d). The dots above the red line are the outliers. The BGCs are colored according to their proposed activity given their similarity with BGCs from MIBiG.
